# Inhibition of T-cell activity in alopecia areata: recent developments and new directions

**DOI:** 10.3389/fimmu.2023.1243556

**Published:** 2023-11-06

**Authors:** Thierry Passeron, Brett King, Julien Seneschal, Martin Steinhoff, Ali Jabbari, Manabu Ohyama, Desmond J. Tobin, Simran Randhawa, Aaron Winkler, Jean-Baptiste Telliez, David Martin, Alexandre Lejeune

**Affiliations:** ^1^ University Côte d’Azur, Centre Hospitalier Universitaire Nice, Department of Dermatology, Nice, France; ^2^ University Côte d’Azur, INSERM, U1065, C3M, Nice, France; ^3^ Department of Dermatology, Yale University School of Medicine, New Haven, CT, United States; ^4^ Department of Dermatology and Paediatric Dermatology, National Reference Centre for Rare Skin Diseases, Saint-André Hospital, University of Bordeaux, Bordeaux, France; ^5^ Bordeaux University, Centre national de la recherche scientifique (CNRS), ImmunoConcept, UMR5164, Bordeaux, France; ^6^ Department of Dermatology and Venereology, Hamad Medical Corporation, Doha, Qatar; ^7^ Translational Research Institute, Academic Health System, Hamad Medical Corporation, Doha, Qatar; ^8^ Dermatology Institute, Academic Health System, Hamad Medical Corporation, Doha, Qatar; ^9^ Department of Dermatology and Venereology, Weill Cornell Medicine-Qatar, Doha, Qatar; ^10^ College of Medicine, Qatar University, Doha, Qatar; ^11^ Department of Dermatology, Weill Cornell Medicine, New York, NY, United States; ^12^ College of Health and Life Sciences, Hamad Bin Khalifa University-Qatar, Doha, Qatar; ^13^ Department of Dermatology, University of Iowa, Iowa City, IA, United States; ^14^ Iowa City VA Medical Center, Iowa City, IA, United States; ^15^ Department of Dermatology, Kyorin University Faculty of Medicine, Tokyo, Japan; ^16^ Charles Institute of Dermatology, UCD School of Medicine, University College Dublin, Dublin, Ireland; ^17^ Pfizer Inc, New York, NY, United States; ^18^ Pfizer Inc, Cambridge, MA, United States; ^19^ Pfizer Inc, Paris, France

**Keywords:** Alopecia areata, autoimmune disease, T cells, T-cell receptor, JAK inhibitor

## Abstract

Alopecia areata (AA) is an autoimmune disease that has a complex underlying immunopathogenesis characterized by nonscarring hair loss ranging from small bald patches to complete loss of scalp, face, and/or body hair. Although the etiopathogenesis of AA has not yet been fully characterized, immune privilege collapse at the hair follicle (HF) followed by T-cell receptor recognition of exposed HF autoantigens by autoreactive cytotoxic CD8^+^ T cells is now understood to play a central role. Few treatment options are available, with the Janus kinase (JAK) 1/2 inhibitor baricitinib (2022) and the selective JAK3/tyrosine kinase expressed in hepatocellular carcinoma (TEC) inhibitor ritlecitinib (2023) being the only US Food and Drug Administration–approved systemic medications thus far for severe AA. Several other treatments are used off-label with limited efficacy and/or suboptimal safety and tolerability. With an increased understanding of the T-cell–mediated autoimmune and inflammatory pathogenesis of AA, additional therapeutic pathways beyond JAK inhibition are currently under investigation for the development of AA therapies. This narrative review presents a detailed overview about the role of T cells and T-cell–signaling pathways in the pathogenesis of AA, with a focus on those pathways targeted by drugs in clinical development for the treatment of AA. A detailed summary of new drugs targeting these pathways with expert commentary on future directions for AA drug development and the importance of targeting multiple T-cell–signaling pathways is also provided in this review.

## 1 Introduction

Alopecia areata (AA) is an autoimmune disease that has an underlying immunoinflammatory pathogenesis, characterized by nonscarring hair loss of the scalp, face, and/or body (1, 2). Hair loss from AA is unpredictable and may relapse and remit or be persistent (2, 3). One or many patches of hair loss may be present, hair loss may be complete on the scalp (alopecia totalis), or there may be complete loss of scalp, facial, and body hair (alopecia universalis) (1). Nail involvement is common, manifesting as pitting and trachyonychia (4). A recent US study reports that AA prevalence in the US has increased from 0.199% in 2016 to 0.222% in 2019 (5), and the global prevalence and lifetime incidence of AA have both been reported to be approximately 2% (6, 7). Both children and adults can be affected, with 31% to 48% of all patients presenting with AA before age 20 years (8, 9). AA can lead to decreased health-related quality of life, and patients frequently find the hair loss and associated stigma to be psychologically and psychosocially burdensome (10–17). Several T-cell–mediated autoimmune comorbidities have been reported to be associated with AA, including vitiligo, Hashimoto thyroiditis, celiac disease, type 1 diabetes, myasthenia gravis, psoriasis, and systemic lupus erythematosus (18–20). Patients with atopic dermatitis (AD) have a 2.98-fold higher risk of developing AA (21). Although the strength of many of these associations has been well established, others, including systemic lupus erythematosus, remain unclear and disputed (22), and some evidence suggests protection from type 1 diabetes among patients with AA (23).

Currently, few treatment options are available (3, 24), with the Janus kinase (JAK) 1/2 inhibitor baricitinib approved for treatment of severe AA in adults and the JAK3/tyrosine kinase expressed in hepatocellular carcinoma (TEC) inhibitor ritlecitinib recently approved for treatment of severe AA in adolescents and adults (25, 26). Other therapies, including glucocorticosteroids, cyclosporine A, methotrexate, and other anti-inflammatory drugs or contact immunotherapy for immunomodulation, are commonly used off-label with varying degrees of efficacy, safety, and tolerability (3, 27, 28). Patients have reported high levels of treatment dissatisfaction due to inefficacy, adverse events (AEs), needle-injection pain, or other intolerabilities (11, 13, 29). The chronic nature of AA, coupled with the potential for repeated relapse of symptoms (30), can lead to exhaustion of currently available therapeutic options. As such, there exists a large unmet medical need for the treatment of AA, particularly for safe, well-tolerated, and effective therapies that can control AA over the long-term.

The underlying immunoinflammatory pathogenesis of AA is complex and not completely characterized, although the collapse of hair follicle (HF) immune privilege and recognition of exposed HF autoantigens via T-cell receptors (TCRs) by autoreactive cytotoxic CD8^+^ T cells is now thought to play a central role (31–35). Extracellular and intracellular signaling pathways regulating T-cell activity may serve as useful therapeutic targets, and several therapies are in development for the treatment of AA. Among these, some targeting JAK signaling (36) have shown efficacy; however, additional related mechanisms of T-cell–mediated AA pathogenesis hold promise as therapeutic targets. The recent approval of ritlecitinib, a selective inhibitor of JAK3 and the TEC family of kinases, underscores the potential role of TCRs and downstream signaling by TEC kinases (26). Other mechanisms currently under investigation and development in AA include pathways that control T-cell recruitment to the HF and upstream cytokine signaling. While not currently under active development by the pharmaceutical industry, other targets related to T cells that are of interest for future development include regulatory T cells (Tregs), immune tolerance, and the microbiome.

The purpose of this review is to provide an overview of T-cell activity and signaling in AA, to explore recent AA therapies targeting T cells, and to provide expert commentary on the importance of targeting multiple T-cell–signaling pathways for the treatment of AA.

## 2 Statement of literature search

Relevant articles were identified by a series of PubMed searches covering publications through March 2023. Representative search strings included “T cells” OR “T cell signaling” OR “T cell receptor signaling” OR “Janus kinase signaling” AND “alopecia areata” OR “therapeutics” OR “drugs.” Additional searches based on authors’ expertise were conducted to identify relevant literature concerning specific pathways of interest, including programmed death 1 (PD-1), TEC family kinase signaling, sphingosine-1-phosphate (S1P) and T-cell migration, Tregs, and autoimmunity and tolerance. Publications mentioning immunopathogenesis of AA, T-cell–signaling pathways in AA, related immune-mediated inflammatory conditions, or therapeutics in development for treatment of AA were considered for inclusion. Publications deemed irrelevant (author replies, errata, etc) were excluded. References cited within relevant articles, as well as any studies previously known to the authors, were included based on the criteria described. Finally, this literature search returned references involving human studies as well as model systems such as the C3H/HeJ inbred mouse model of AA. Systemic drugs currently undergoing clinical trials for the treatment of AA were retrieved from ClinicalTrials.gov as of April 28, 2023, using criteria for recruitment status (recruiting, not yet recruiting, active; not recruiting, enrolling by invitation studies), disease (“alopecia areata”), and funding (industry). The therapeutic pipelines of identified companies were searched to ensure active and ongoing clinical development.

## 3 T cells and the pathophysiology of AA

### 3.1 HF immune privilege and loss thereof in AA

The lower epithelium of the healthy anagen HF is an immune-privileged site, as several mechanisms decrease the number of distributing immunocytes around the HF, thus preventing infiltration and activation of potentially autoreactive T cells and subsequent autoimmune response (2, 37). Several mechanisms are thought to be responsible for this immune privilege, including physical barriers to lymphocyte infiltration (38), downregulation of major histocompatibility complex (MHC) I (2, 39–41), localized expression of immunosuppressants (including transforming growth factor [TGF]-β, α-melanocyte-stimulating hormone [α-MSH], interleukin (IL)-10, macrophage migration inhibitory factor (MIF), and somatostatin) (2, 38, 40, 41), low MHC I chain-related protein A (MICA) expression (39, 40), maintenance and distribution of memory T cells (42), and downregulation of natural killer group 2 member D (NKG2D) receptor on local NK cells (41). Breakdown of this HF immune privilege is thought to be essential for the onset of AA (31), likely occurring when the balance of signaling pathways upholding this immune privilege is overwhelmed by those leading to collapse, including interferon (IFN)-γ–induced upregulation of MHC classes I and II antigen-presenting molecules, loss of local TGF-β–dependent immunosuppression, increased MICA expression, decreased MIF expression, and mast cell degranulation (43). The relative contribution, triggers, and chronological sequence of each of these events related to immune privilege collapse in the context of AA remain to be established. Regardless of cause, the collapse of immune privilege leaves the HF exposed to immune surveillance and is followed by antigen-presenting cells that activate autoreactive T cells and the recognition of exposed HF autoantigens by lymphocytes (31).

**Figure 1:**
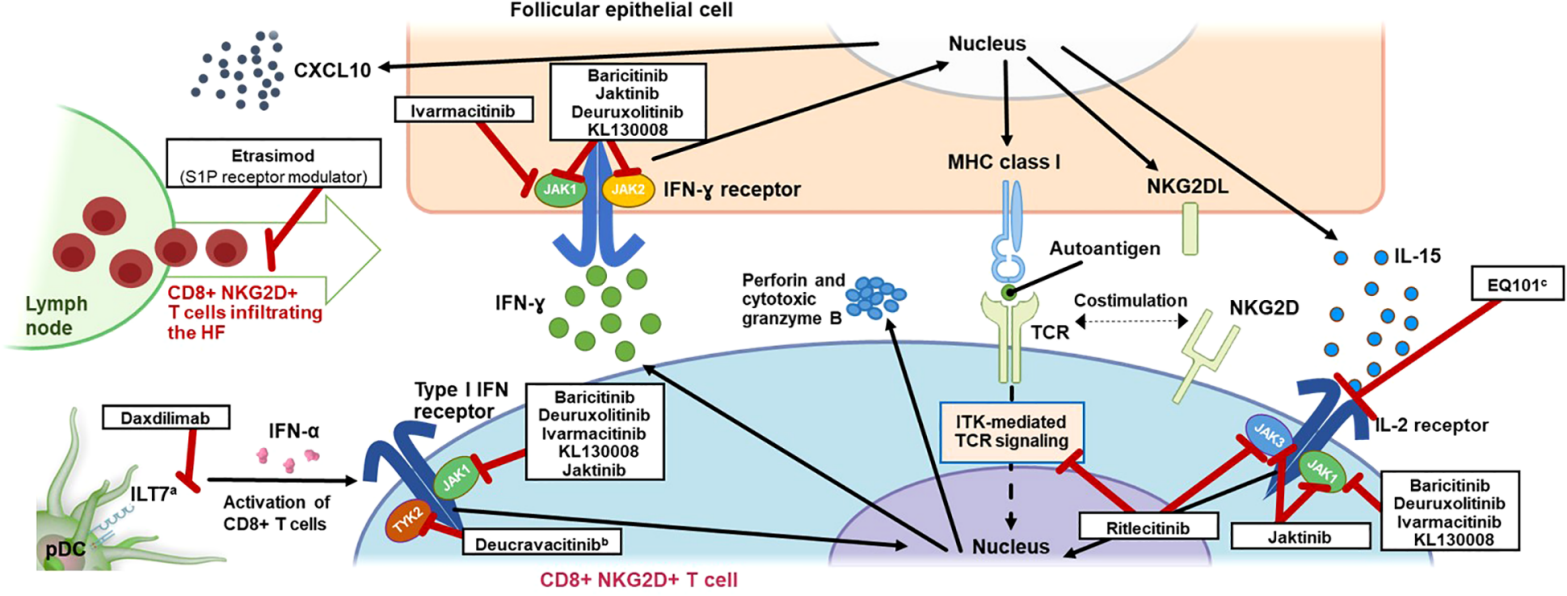
Mechanism of Action of T-Cell Signaling in AA and Inhibitors Targeting Key Pathways. AA, alopecia areata; CD, cluster of differentiation; CXCL10, C-X-C motif chemokine ligand 10; HF, hair follicle; IFN, interferon; IL, interleukin; ILT7, leukocyte immunoglobulin-like receptor subfamily A member 4; ITK, interleukin-2–inducible T-cell kinase; JAK, Janus kinase; MHC, major histocompatibility complex; NKG2D(L), natural killer group 2 member D (ligand); pDC, plasmacytoid dendritic cell; S1P, sphingosine-1-phosphate; TCR, T-cell receptor; TYK2, tyrosine kinase 2. ^a^ ILT7 is activated by bone marrow stromal cell antigen 2 (BST2*)*, a surface-expressed protein upregulated in cells exposed to INF-α, leading to a negative feedback loop resulting in restricted production of INF-α by pDCs (63). ^b^ Inhibition of TYK2 by deucravacitinib also inhibits differentiation of T cells via pDC-derived IL-23. ^c^ EQ101 (formerly known as BNZ-I) also inhibits IL-2 and IL-9 signaling.

Skin biopsies from patients with AA show a perifollicular infiltrate of antigen-presenting cells and T cells (CD4^+^ and CD8^+^) and elevated expression of MHC class I (and class II) molecules (39). A genome-wide association study (44) and studies implicating specific HLA class I and class II alleles in AA (45–47) support the centrality of T-cell responses in AA pathophysiology. Indeed, AA is thought to be mediated by CD4^+^ T cells and cytotoxic CD8^+^ T cells in and around HFs (36, 48). Additionally, γδ T cells have been shown in humans to induce the pathophysiological hallmarks of AA and have been identified around nonlesional AA HFs (49, 50), suggesting a potential role in the early stages of AA pathogenesis. This infiltration in and around the HF has been coined a “swarm of bees” and primarily consists of T cells but also NK cells, eosinophils, plasmacytoid dendritic cells (pDCs), mast cells, and macrophages (51, 52). Because immune cells transit into skin via the vascular system, there may be a role for S1P and C-X-C motif chemokine receptor 3A (CXCR3A) signaling in mediating the cellular infiltrate (53–55). The persistence of CD8^+^ T cells around the lower anagen HF may contribute to prolonged hair loss in chronic and/or severe AA by promoting the HF to enter the telogen phase and preventing the empty or “kenogen” HF from reentering the anagen phase (52); further, the presence of CD8^+^ T cells may indicate that specific chemokines drive AA pathogenesis. While the relative contribution of skin resident T cells over those newly infiltrated in the pathogenesis of AA is not known, several studies have reported expression of chemokines in AA and their contribution to HF immune infiltration (53, 56, 57). Briefly, CXCL9, CXCL10, and CXCL11 are upregulated in AA lesions along with their common receptor CXCR3, inhibition of CXCR3 in a mouse model of AA prevented onset of disease (53), and CXCL10 has been suggested to induce the infiltration of Th1 and Tc1 cells (57). CCL13 upregulation has also been demonstrated in AA lesions (56). Additionally, lesional CD8^+^ T-cell numbers are high in alopecia totalis and alopecia universalis (1). Foxp3^+^ Tregs may contribute to the amelioration of AA through maintenance of HF immune privilege (31), and dysregulation or impaired function of Tregs has been implicated in AA in animal and human studies (44, 58–61); further, relatively few Tregs have been shown to infiltrate the HF in AA (62). During AA pathogenesis, various cytokines are produced and a complex and multifactorial signaling response occurs (**Figure 1**). Other contributors include strong expression of NKG2D, a type II transmembrane receptor involved in the killing response (64), on infiltrating CD8^+^ T cells and NK cells; overexpression of activating NKG2D ligands (UL16-binding protein, MICA) on HFs; and downregulation of immunosuppressants (31). Tissue resident memory T cells have been hypothesized to be involved in AA pathogenesis and lesion recurrence (65). This notion is based on increased levels of these cells in AA lesions observed in some studies (65), including evidence from AA mouse models linking injection of high proportions of effector memory CD8+ T cells with increased incidence of AA development (66). More recently, a CD44^super-high^Cd49d^low^ subset of CD8^+^ virtual memory T (T_vm_) cells have been identified in stomach-draining lymph nodes of alopecic mice with the ability to promote disease upon transfer to naive recipient mice. These cells differentiated from conventional T_vm_ cells via IL-12, IL-15, and IL-18 stimulation and were capable of NKG2D-dependent cytolytic activity without TCR activation (67). Interestingly, heterogeneous populations of CD8^+^ T cells were described in AA showing a gradual continuum of effector functions and homing instead of clearly differentiated clusters of cells (68). Importantly, IL-7 and IL-15 derived from hair follicles can regulate the tissue residence of skin-resident memory T cells. The AA-implicated cytokine IL-15 discussed below is important for the differentiation of tissue resident memory T cells (69, 70) and, along with IL-7, has been shown to regulate tissue residence of these T cells (71).

### 3.2 IFN-γ–IL-15 positive feedback loop and the JAK/STAT pathway in AA

IFN-γ is known to be a key driver of AA pathogenesis (72), although mechanistic questions remain regarding early AA. These include the relative timing of IFN-γ increases and immune privilege collapse at the HF and any requisite threshold concentration of IFN-γ that may trigger AA. IFN-γ levels are indeed higher in the blood of patients with AA than those without (73, 74), and one study has shown a positive correlation with disease severity (73). Additionally, results from the C3H/HeJ mouse model suggest that inhibition or deficiency of IFN-γ can prevent the development of AA (36, 75). Although adaptive CD8^+^ T cells have been considered the primary drivers of AA, additional sources of IFN-γ in AA have recently been shown to include type 1 innate lymphoid cells, NK cells, invariant NK T cells, and γδ T cells (42), any of which could potentially serve as initiators and/or drivers of AA pathogenesis. Some evidence from C3H/HeJ AA mice suggests that pDC-dependent release of IFN-α at the HF may exist upstream of other mechanisms of AA pathogenesis, including IFN-γ release and collapse of HP immune privilege leading to AA (76). IFN-α release from pDCs could itself be stimulated by innate immunity, including Toll-like receptor (TLR)–mediated processes, potentially as a result of viral infection (77).

Once AA pathogenesis is triggered, the mechanistic role of IFN-γ is more clearly understood. Binding to IFN-γ receptors and subsequent signaling along the T helper (Th)1 axis through nonreceptor tyrosine kinases JAK1/JAK2 may contribute to immune privilege collapse via upregulation of MHC class I and NKG2D receptor expression (2, 39, 78). This in turn may lead to activation of autoreactive CD8^+^ T cells via presentation of HF autoantigens, represented potentially by trichohyalin peptides (33) and other autoantigens, by MHC class I molecules (39). IFN-γ also stimulates the production of IL-2 and IL-15; activated CD8^+^ T cells stimulated by IL-15 further mediate IFN-γ secretion via the JAK/signal transducer and activator of transcription (STAT) pathway (36), specifically through JAK1/JAK3 signaling (79, 80).

IL-15 has been implicated in the pathogenesis of AA: in the C3H/HeJ inbred mouse model of AA, disease development was prevented with an anti–IL-15 receptor antibody (36); IL-15 is elevated in the serum of patients with AA (81–84), and increased expression of IL-15 and the IL-15 receptor (IL-15Rα) in NKG2D^+^CD8^+^ T cells and around lesional scalp HFs has also been identified at the mRNA level (36, 85). Data from clinical trials of JAK inhibitors discussed later also emphasize the key role of IL-15 signaling in AA. This intracellular signaling consisting of IFN-γ and IL-15 creates a JAK/STAT-dependent cytokine loop that may contribute to autoimmunity and sustained inflammation in AA (**Figure 1**). Nevertheless, the contribution of this inflammatory loop at various clinical stages and chronicity of the disease needs to be further investigated.

Other cytokines that bind to the common γ-chain cytokine receptor and signal through JAK1/JAK3 may play a role in the pathogenesis of AA. Two studies using the C3H/HeJ mouse model of AA suggest that IL-2 (36) and IL-7 (86), both signaling through JAK1/JAK3, may partially contribute to the development of AA pathology (36, 86). Through interaction with the common γ-chain cytokine receptor, additional cytokines such as IL-4, IL-9, and IL-21 are mechanistically involved in immune system homeostasis, including T-cell development and differentiation (87, 88). As such, these cytokines may contribute to AA pathophysiology, although investigation is needed.

**Figure 2:**
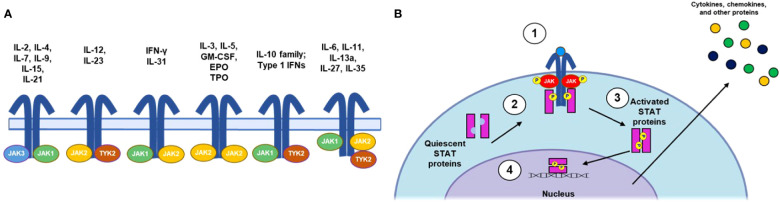
Summary of JAK/STAT Signaling Pathways. **(A)** IL receptor-JAK protein configurations. **(B)** Canonical JAK/STAT signaling mechanism: (1) the cytokine binds to and induces association of receptor subunits; (2) receptor-associated JAKs become phosphorylated and subsequently phosphorylate the intracellular tail of the cytokine receptor; (3) phosphorylation allows docking of STATs to the receptors, where they become phosphorylated; and (4) phosphorylated STATs dimerize and translocate to the nucleus, where they regulate downstream transcription of inflammatory factors. EPO, erythropoietin; GM-CSF, granulocyte-macrophage colony-stimulating factor; IFN, interferon; IL, interleukin; JAK, Janus kinase; NK, natural killer; STAT, signal transducer and activator of transcription; TPO, thrombopoietin; Th, T helper cell; TYK, nonreceptor tyrosine kinase.

Genome-wide association studies in patients with AA (44, 89) and the confirmation that IFN-γ signals through JAK1/2 and IL-15 via JAK1/3 (36, 90) provided initial rationale for developing JAK inhibitors in AA. Consisting of JAK1, JAK2, JAK3, and tyrosine kinase 2 (TYK2) (91), the JAK family of nonreceptor tyrosine kinases mediates intracellular signaling through the JAK/STAT pathway (**Figure 2**) (92). JAKs associate with distinct subunits of JAK-dependent cytokine receptors (93) and are phosphorylated upon cytokine binding to the receptor complex. Subsequent phosphorylation of the receptor by JAKs leads to the recruitment and phosphorylation of STATs, which can homo- or heterodimerize and translocate to the nucleus, where they regulate gene transcription. Importantly, specific cytokines can activate various JAKs and STATs in combination, leading to different cellular responses (92, 94). Indeed, JAK/STAT signaling has proven to be an early target for development of therapeutics for AA and has been extensively reviewed elsewhere with respect to both general immunology (92) and AA specifically (95, 96).

### 3.3 Th2 and Th17 axis cytokines

In addition to the Th1/IFN‐γ axis, the Th2 axis has been suggested to play a role in AA pathophysiology as well (97, 98). Levels of IL-4, IL-13, chemokine ligand 18 (CCL18), and thymic stromal lymphopoietin (TSLP) have been found to be elevated in AA scalp lesions, with levels of IL-4, IL-5, IL-6, CCL17, and immunoglobulin E (IgE) elevated and eosinophilia more prevalent in the serum/blood of patients with AA (97). In a separate study, CCL13, CCL17, CCL22, CCL26, and IL-13 expression was observed in AA, with a positive association between AA severity and levels of both CCL13 and IL-13 (99). Serum CCL17 has been proposed as a disease activity and treatment response biomarker, as levels have been found to be elevated with increasing disease severity and among poor vs adequate responders to intravenous corticosteroid pulse therapy (100). Observations from clinical cases and a phase 2a clinical trial provide conflicting data regarding IL-4 and IL-13, with some patients developing new-onset AA after initiation of dupilumab, an IL-4/13 blocker, for treatment of atopic dermatitis and other patients with AA experiencing hair regrowth with dupilumab treatment (101, 102). Interestingly, patients with higher baseline IgE levels experienced greater response to dupilumab (102), suggesting that an atopic background could be associated with some response to IL-4 and IL-13 inhibition among patients with AA (103). Mice overexpressing IL-31 spontaneously not only develop atopic dermatitis but also histologically proven alopecia areata (104). Additionally, atopy itself commonly occurs among patients with AA (21, 105) and has been traditionally identified as a risk factor and negative prognostic marker for AA (106). However, recent evidence suggests that the use of JAK inhibitors when treating patients with an atopic background may offer a positive prognosis for future development of AA (107).

**Figure 3:**
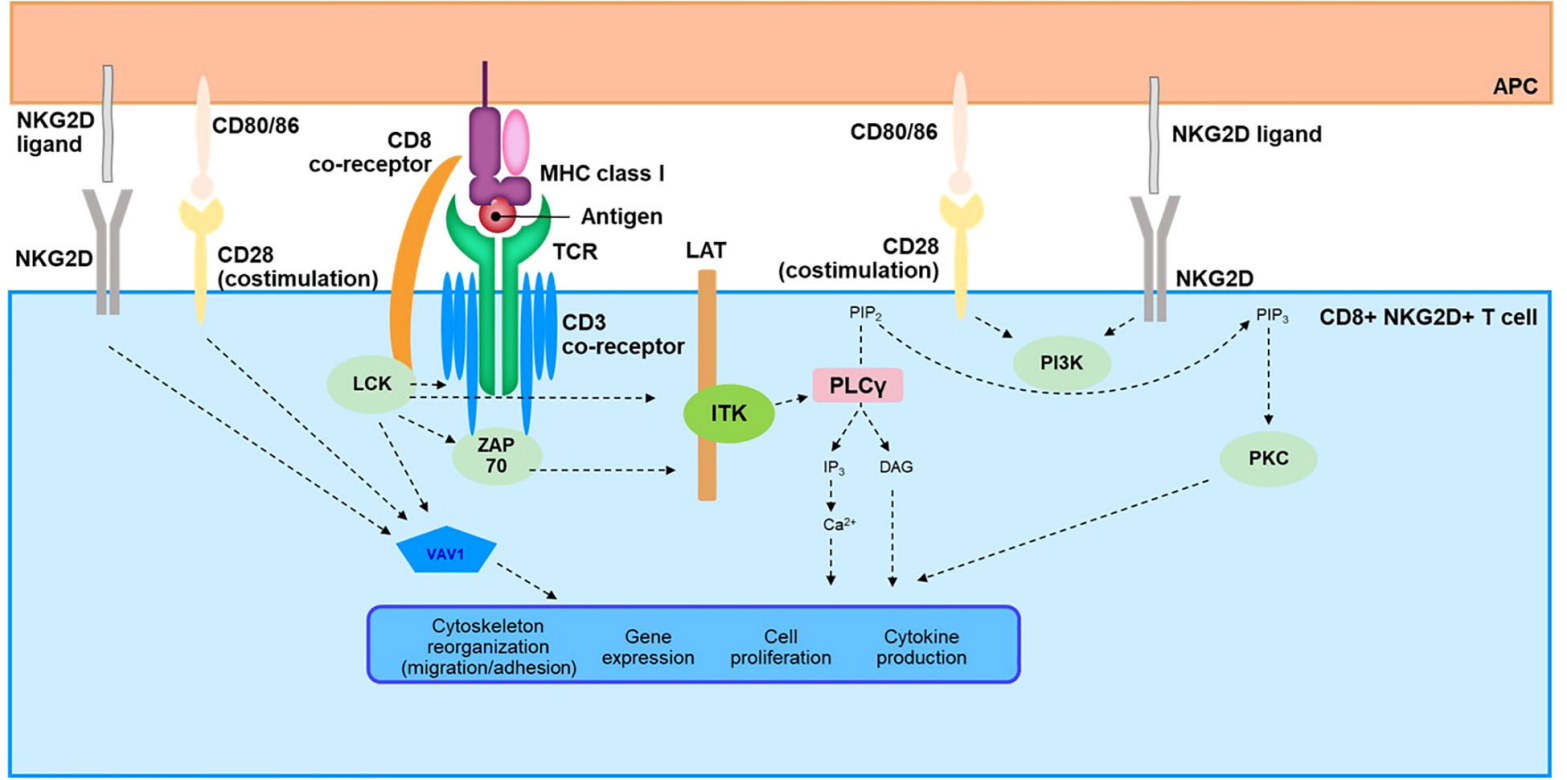
Overview of TCR Signaling Cascade in CD8^+^NKG2D^+^ T Cells. APC, antigen-presenting cell; Ca, calcium; CD, cluster of differentiation; DAG, diacylglycerol; IP_3_, inositol-1,4,5-triphosphate; ITK, interleukin-2–inducible T-cell kinase; LAT, linker for activation of T cells; LCK, lymphocyte-specific protein tyrosine kinase; MHC, major histocompatibility complex; NKG2D, natural killer group 2 member D; PIP_2_, phosphatidylinositol 4,5-bisphosphate; PIP_3_, phosphatidylinositol-3, 4, 5-triphosphate; PKC, protein kinase C; PLCγ, phospholipase C γ; SLP76, SRC-homology-2-domain-containing leukocyte protein of 76 kDa; TCR, T-cell receptor; ZAP70, zeta chain–associated protein kinase 70.

Levels of Th17-related cytokines, including IL-17 and IL-22, have also been reported to be elevated in AA lesions (108). IL-17–producing cells have been found to be densely infiltrated around AA HFs (62), and serum IL-17 levels are elevated in patients with AA (60, 108–111). Furthermore, serum and lesional IL-17 levels have been shown in a few studies to positively correlate with AA severity (108, 109). However, in a small study of patients with AA, most did not experience amelioration of AA upon inhibition of IL-17A, and worsening of hair loss was observed in some (112). Inhibition of IL-12/23 signaling, which supports Th17 cell development, was not observed to improve AA in a study of C3H/HeJ mice and human patients (113). Additionally, some new cases of AA have been observed to occur among patients receiving IL-12/23 inhibitor treatment for other indications (114, 115). Although IL-17 primarily signals through a JAK-independent pathway (116), most of the cytokines described here signal through JAKs (**Figure 3**) (92, 94).

### 3.4 TCR and its signaling pathway

Although self-antigens remain largely uncharacterized in AA, peptides derived from melanocytes and keratinocytes may be candidates (33, 35). Among these, peptides from trichohyalin and tyrosinase-related proteins were shown to induce a higher proportion of cytotoxic T lymphocytes releasing IFN-γ in AA compared with healthy peripheral blood mononuclear cells, suggesting the presence of autoreactive cells against these antigens in AA (33). This evidence supports the recognition of autoantigen-MHC complexes by TCRs of autoreactive CD4^+^ and CD8^+^ T cells as an important step in activation of these immune cells and ultimately disease pathophysiology (**Figure 1**). It also implies activation of the signaling pathway downstream of the TCR.

The TCR represents a complex signaling pathway that has been reviewed in detail elsewhere (117, 118). Here, we provide a brief overview to facilitate understanding of possible TCR signaling in AA pathogenesis (**Figure 3**). The TCR lacks intrinsic catalytic activity and relies on a complex downstream cascade involving several kinases, scaffold proteins, and second messengers (118, 119). The 2 most proximal kinases, lymphocyte-specific protein tyrosine kinase (LCK) and zeta chain–associated protein kinase (ZAP70), are key components that initiate this transduction pathway (120–122) and lead to phosphorylation of linker for activation of T cells (LAT), a scaffold protein (123, 124). Phosphorylated LAT recruits several mediators to form a signalosome, including adaptors Grb2-related adaptor downstream of Shc (GADS) and SH2 domain–containing leukocyte protein of 76 kDa (SLP76), and the kinase IL-2–inducible T-cell kinase (ITK), a member of the TEC family of kinases (125–128). ITK then activates phospholipase C γ1 (PLC-γ1) by phosphorylation, leading to enzymatic production of the second messengers inositol triphosphate (IP_3_) and diacylglycerol (DAG) (119, 129). IP_3_ and DAG are key effectors for activation of downstream cellular pathways orchestrated by calcium influx and transcription factors such as nuclear factor of activated T cells (NFAT), nuclear factor κB (NF-κB), and activator protein-1 (AP-1) (119, 130). Upon activation of the TCR, other cellular effectors, such as RhoGTPase, are also engaged through the guanine nucleotide exchange factor VAV1 and the phosphoinositide 3-kinase (PI3K)/mechanistic target of rapamycin (mTOR) pathways (118). Ultimately, the TCR signalosome leads to critical functions of T cells, including production of cytokines and proliferation and trafficking of these immune cells through regulation of cytoskeletal dynamics and modulation of gene expression (119, 130–132).

In AA, the TCR signaling pathway remains largely unexplored despite the role of autoreactive T cells in the pathophysiology of the disease. Two studies have nevertheless shown data directly supporting activation in patients with AA (133, 134). Pathway analysis of upregulated genes in AA scalp biopsy compared with those in healthy controls identified the TCR signaling pathway as one of the most enriched pathways (133). A study using exogenously stimulated T cells isolated from skin punch biopsies of patients with AA revealed quantitative changes in the TCR-signaling network and activation of this pathway. The TCR/CD3 receptor complex; most kinases involved in the downstream signaling such as LCK, ZAP70, and ITK; and the scaffold protein LAT and adaptors GADS and SLP76 were all shown to be upregulated (134).

The activation of the TCR signaling pathway by HF autoantigens in T cells implies a breach of immune tolerance. Although the mechanisms underlying immune privilege collapse have been documented (31, 32), the breach of tolerance at the level of T cells needs to be further explored. Indeed, the likely presence of low to intermediate levels of peripheral autoreactive T cells that escape negative selection in the thymus is well established in healthy individuals. However, various mechanisms have evolved in the immune system to protect against autoimmunity under steady-state conditions. Both immune tolerance and autoreactive T cells have been reviewed in the context of inflammatory skin diseases, including AA (37). Nevertheless, recent findings could help to understand the disruption of peripheral immune tolerance in AA in the context of immune privilege collapse. Three general concepts related to TCR signal strength are important to determine the fate of T cells, including tolerance in autoreactive T cells: TCR affinity, avidity, and functional avidity (135). Therefore, modification in these 3 components of TCR signaling could contribute to disruption of immune tolerance, leading to autoimmunity.

The first concept, affinity, describes the strength of the interaction between a single TCR and a peptide-MHC complex. It has been suggested that posttranslational modifications of HF autoantigens in AA lead to increased binding affinity to MHC (35). Particularly, trichohyalin has been proposed as a promising candidate due to its expression at the early stage of the anagen phase; additionally, it is subject to citrullination and deamidation (35), 2 posttranslational modifications that can affect binding affinity.

The second characteristic, TCR avidity, is the total strength resulting from multiple TCR-MHC engagements. It is dependent on the concentration of TCR, MHC, and antigen. In AA, increased expression of MHC I and MHC II upon release of IFN-γ, as well as ectopic expression of HF antigens, are well-established key contributors to HF immune privilege collapse (31, 32). Therefore, immune privilege collapse likely results in changes to TCR avidity from autoreactive T cells against their potential autoantigens, impacting TCR signal strength.

Finally, functional avidity integrates not only TCR avidity against antigen-MHC complexes but also concomitant signals that can influence TCR downstream signaling. Such signals include co-stimulatory/inhibitory receptors and ligands, cytokine milieu, and adhesion molecules that can modify the immune synapse (135). Studies in AA have reported changes occurring at the level of functional avidity, therefore suggesting a critical role in immune tolerance maintenance and disruption. Co-inhibitory signals such as PD-1/programmed death ligand 1 (PD-L1) or cytotoxic T-lymphocyte–associated protein 4 (CTLA4) are expressed in HF cells and therefore are likely involved in immune privilege to maintain tolerance (136, 137). Both pathways, known as immune checkpoints, modulate and inhibit the TCR signaling network through various mechanisms like change of phosphorylation status of downstream mediators (138). In contrast, co-stimulatory signals of T cells contribute to immune privilege collapse. In a mouse model of AA, the co-stimulatory receptor CD28 and its cognate ligands CD80 and CD86, as well as CD40 and CD40L, were significantly upregulated (139); all are known contributors to T-cell activation. Signaling cascades engaged by both receptors share common cellular effectors with the TCR pathway such as PI3K, as well as VAV1 and JAK3, which can also be activated downstream of CD28 and CD40, respectively (140, 141). Recently, CD28 was described as a potential biomarker for progression of AA to alopecia totalis or alopecia universalis (133). Published literature has also highlighted the role of the NKG2D receptor in the pathophysiology of AA. NKG2D expressed on NK and T cells recognizes cell surface ligands broadly induced by cellular stress and relies on the adaptor protein DAP10 for downstream signaling (64, 142). While engagement of this receptor is sufficient to induce activation of NK cells, the main body of evidence suggests that it acts as a costimulatory signal together with the TCR to promote activation of T cells, likely through modulation of common shared pathways involving PI3K and Grb2-VAV (64, 143, 144). Studies have also reported NKG2D-induced cytolytic activity in T cells independent of TCR engagement upon IL-15 stimulation and phosphorylation of DAP10 by JAK3 (64, 145). Evidence of a critical role in AA comes both from mouse models showing CD8^+^NKG2D^+^ T cells to be necessary and sufficient to induce the disease (36), as well as a genome-wide association study in patients with AA showing association with activating ligands of the NKG2D receptor (44). Interestingly, a role has been suggested for this pathway in autoimmune diseases, such as vitiligo and AA, by reducing the threshold for T-cell activation via the TCR (64). This contribution of NKG2D to the pathogenesis of vitiligo was based on strong induction of CD8^+^ T-cell response against a melanocyte antigen common with AA in mouse autoimmune vitiligo (146). Finally, integration of TLR signaling at the level of the TCR downstream cascade should receive further attention in AA, as co-stimulatory interaction has been described for these pathways in viral infection (147). Evidence exists regarding involvement of TLRs in AA, both at the level of genetic polymorphism (148) as well as gene and protein upregulation (149, 150); therefore, TLRs could serve as additional enhancers of the TCR signal.

Regarding the role of cytokines in functional avidity, a vast literature has described elevation of the levels of these immune mediators in AA, as previously discussed in this review. Amongst them, higher serum levels of IL-2 and IL-12 (84, 98) are probably critical due to their important contribution to the differentiation of CD8^+^ T cells into effector cells (151, 152). Low-dose IL-2 can exert an opposite effect by promoting immunosuppression through differentiation of Tregs. The use of this cytokine has been explored as a potential therapeutic in various autoimmune conditions (153). However, this approach in both the C3H/HeJ mouse model of AA (154) and in a placebo-controlled trial in patients with moderate to severe AA (155) failed to show clear positive improvement, leading to questions about the use of low-dose IL-2 to achieve therapeutic benefit from Treg-mediated immunosuppression and possibly the overall contribution of Treg cells in this disease. Finally, decreased expression of immunosuppressive cytokines such as TGF-β1/2 and IL-10 occurring during HF immune privilege collapse may contribute to activation of autoreactive T cells (31). Although JAK inhibitors for AA may contribute to inhibition of IL-10 and TGF-β1/2 signaling, no drugs specifically targeting these cytokines are currently in development for AA.

Downstream consequences of perturbations to the TCR signaling concepts described here hold profound consequences for regulation of gene expression and cell migration, growth, and activation (118, 119). These processes contribute to the pathophysiology of AA and could represent 1 or more targets for therapeutic development.

## 4 Systemic therapies targeting T cells under development for AA

To date, only 2 oral systemic therapies have been approved for the treatment of AA; these include the JAK1/2 inhibitor baricitinib, which has been approved in several countries and regions including the US and Europe (25), and the selective JAK3/TEC kinase inhibitor ritlecitinib, which is also approved in the US and Europe (26). These and several systemic targeted therapies currently under development as identified from ClinicalTrials.gov are reviewed here (**Figure 1**). The amount of data published in the literature is highly variable between identified treatments. When available, preclinical and clinical data regarding the mechanism of action are discussed, as well as a brief overview of the efficacy and safety from published clinical trials of patients with AA. It should be noted that not all patients improve in response to the inhibitors discussed below, and relapse is likely upon treatment discontinuation.

### 4.1 JAK inhibitors

JAK inhibitors function by suppressing cytokine signaling through the JAK/STAT pathway, as discussed earlier (**Figure 2**) (93, 156), and several JAK inhibitors with various profiles of selectivity toward JAK isoforms are currently under development for AA (95). The only JAK3 selective inhibitor approved or under investigation for treatment of AA, ritlecitinib, will be discussed separately due to a dual mechanism of action targeting the TEC family kinases.

Baricitinib is an orally administered, selective, and reversible noncovalent inhibitor of JAK1 and JAK2 that was recently approved to treat adults with severe AA (25, 157). Baricitinib was previously approved for the treatment of moderate to severe active rheumatoid arthritis in adults (158), is approved by the US Food and Drug Administration (FDA) for hospitalized adults with COVID-19 (159, 160), and is approved by the European Medicines Agency (and currently under FDA review) for moderate to severe atopic dermatitis in adults (161).

In preclinical studies, baricitinib reduced both infiltration of CD8^+^NKG2D^+^ cells and MHC class I and class II expression in C3H/HeJ AA mice vs control treatment (162). Gene expression profiling found that baricitinib induced rapid normalization of the expression signature of both type I and type II IFN in this mouse model (162). Importantly, the overall IFN and cytotoxic T-lymphocyte components of the Alopecia Areata Disease Activity Index (ALADIN) (36) could discriminate between mice experiencing disease resolution and those without resolution after baricitinib treatment (162).

Two phase 3 studies, BRAVE-AA1 (NCT03570749) and BRAVE-AA2 (NCT03899259), investigated the efficacy and safety of baricitinib in adult patients with ≥50% scalp hair loss (25). The primary endpoint for both studies was the proportion of patients with a Severity of Alopecia Tool (SALT) score of ≤20 (≤20% scalp without hair) at week 36. In BRAVE-AA1, 38.8% of patients receiving baricitinib 4 mg and 22.8% of those receiving baricitinib 2 mg had a SALT score of ≤20 at week 36, compared with 6.2% of patients receiving placebo; efficacy results were similar in BRAVE-AA2, with 35.9%, 19.4%, and 3.3% of patients receiving baricitinib 4 mg, baricitinib 2 mg, and placebo, respectively, with a SALT score of ≤20 at week 36. AEs more common among patients receiving baricitinib than those receiving placebo included acne, urinary tract infection, and elevated levels of creatine kinase. Serious infections, herpes zoster infections, major adverse cardiovascular events (MACE), and malignancies were infrequent, and the overall benefit-risk profile supported approval in AA. The efficacy of baricitinib was sustained through 52 weeks of treatment, and the safety profile was similar to that reported through 36 weeks (163). A phase 3 trial investigating the efficacy, safety, and pharmacokinetics of baricitinib in pediatric patients aged 6 to <18 years is currently recruiting (BRAVA-AA-PEDS; NCT05723198). The primary outcome measure is the proportion of patients with SALT score of ≤20 at week 36.

Deuruxolitinib (formerly CTP-543), a deuterated analogue of ruxolitinib, is an orally administered selective inhibitor of JAK1 and JAK2. Deuteration of small-molecule drugs can modulate pharmacological properties, although the impact of such modification has been variable (164). No study has been identified in the literature regarding the pharmacokinetic or pharmacodynamic implications of deuteration of ruxolitinib. However, studies of ruxolitinib remain informative when the mechanism of action of deuruxolitinib is considered. In preclinical studies, ruxolitinib was found to downregulate MHC class II expression in cultured murine hair bulbs and around HFs after IFN-γ exposure and to stimulate anagen reentry–related molecules in cultured human dermal papilla cells via the Wnt/β-catenin pathway (165). Ruxolitinib was also observed to promote T-cell exhaustion by increasing expression of PD-1 and transcription factors thymocyte selection–associated HMG box and eomesodermin (166). In an open-label clinical study of 12 patients with moderate to severe AA treated with ruxolitinib 20 mg twice daily, changes in gene expression from baseline to week 12 were observed, and the ALADIN IFN and cytotoxic T-lymphocyte components could discriminate eventual responders from non-responders at baseline (167).

Three phase 2 studies of deuruxolitinib in patients with AA have been completed to date (NCT03941548, NCT03811912, and NCT03137381). In the phase 2, double-blind, placebo-controlled, dose-ranging study (NCT03137381), 149 adult patients with AA and ≥50% scalp hair loss were randomized to deuruxolitinib 4 mg, 8 mg, or 12 mg twice daily (168). The primary endpoint was met for the 8- and 12-mg doses, with 47% and 58% (*P*<0.001 vs placebo) of patients exhibiting ≥50% change from baseline SALT score at week 24, respectively (168). Deuruxolitinib was generally well tolerated; the most common treatment-emergent AEs included headache, nasopharyngitis, upper respiratory infection, and acne (168). Two randomized, double-blind, placebo-controlled, phase 3 trials evaluating the efficacy and safety of oral deuruxolitinib in adult patients with AA and ≥50% scalp hair loss have been completed but are not yet published (THRIVE-AA1 [NCT04518995] and THRIVE-AA2 [NCT04797650]). Both trials are designed to evaluate scalp hair regrowth after 24 weeks of treatment with deuruxolitinib 8 or 12 mg twice daily. The primary endpoint in each trial is the percent of patients with a SALT score of ≤20 at week 24. Topline results from THRIVE-AA1 indicate that the primary efficacy endpoint was met (169).

Jaktinib is a deuterated analogue of the JAK inhibitor momelotinib and is reported to be a stronger inhibitor of JAK2 and TYK2 compared with JAK1 (170). Like its parent compound momelotinib, jaktinib can also inhibit activin receptor–like kinase inhibitor 1 (ACVR1), a receptor of bone morphogenic proteins (170). It has been suggested that ACVR1 inhibition improves anemia in myelofibrosis (171), an indication for which momelotinib is approved. Preclinical data suggest that this improvement in anemia could be attributed to a change in expression of hepcidin in the liver, subsequently affecting iron metabolism (171). A search of the literature failed to identify any preclinical or translational data for either jaktinib or momelotinib in AA. A similar mechanism of action as other JAK inhibitors could be expected, although a study of the impact of ACVR1 inhibition in the context of AA would be valuable. Although a phase 1 study showed stable pharmacokinetic properties of jaktinib, no comparison was made with the nondeuterated parent compound, precluding any conclusion regarding potential benefit of this chemical modification (172).

Jaktinib is currently under investigation as a topical cream (jaktinib hydrochloride) in a dose-escalation/dose-extension (phase 1/2), randomized clinical trial in adults with AA (NCT04445363). The primary endpoint of the trial is the proportion of patients with 90% improvement in SALT score at week 24. A phase 2 trial evaluating the 24-week safety and efficacy of jaktinib formulated as an oral tablet in the treatment of adults with ≥50% scalp hair loss due to AA was recently completed (NCT04034134). A phase 3 trial evaluating the safety and efficacy of oral jaktinib in approximately 420 adults with AA and ≥50% scalp hair loss is currently recruiting, with a primary endpoint of SALT score ≤20 response at week 24 (NCT05051761).

Ivarmacitinib (formerly SHR0302) is a selective, oral JAK1 inhibitor under investigation for the treatment of several immunoinflammatory diseases, including AA. The phase 2 CRYSTAL2 (NCT04346316) study was a double-blind, randomized, placebo-controlled, dose-ranging trial to evaluate the safety and efficacy of SHR0302 in adult patients with AA. Patients (n=94) were randomized to ivarmacitinib 2 mg, 4 mg, or 8 mg or placebo, and the primary endpoint was percent change from baseline in SALT score at week 24 (173). Patients receiving any dose of ivarmacitinib achieved greater least squares mean change from baseline in SALT score than patients receiving placebo (ivarmacitinib: 2 mg, −30.5%; 4 mg, −56.1%; 8 mg −51.0%; placebo: −19.9%). Reported SAEs included 1 instance each of COVID-19 pneumonia and follicular lymphoma. In a public communication, the investigating company has stated that a phase 1b trial evaluating the safety, tolerability, and pharmacokinetics of a topical formulation of ivarmacitinib in patients with AA is currently ongoing. No preclinical and translational studies of ivarmacitinib relevant to AA have been published.

KL130008 is a selective oral JAK1/2 inhibitor currently under investigation for AA. In a phase 1 trial of healthy Chinese patients, KL130008 administered in single or multiple ascending doses for 7 days resulted in dose-dependent inhibition of IL-6–induced STAT3 phosphorylation in leukocytes isolated from patients (174). Treatment-emergent AEs occurring with KL130008 but not placebo included grade 1 or 2 decreases in neutrophil percentage, decrease in neutrophil count, and increase in lymphocyte percentage. A phase 2 study of the safety and efficacy in adults with AA, including alopecia totalis or alopecia universalis, and ≥50% scalp hair loss is currently planned (NCT05496426). The primary endpoint will be SALT score ≤20 response at week 24. To date, no preclinical or translational studies of KL130008 relevant to AA have been published.

Deucravacitinib is an allosteric inhibitor of TYK2 (175, 176), a JAK family kinase responsible for mediating type I IFN response, including signaling by IFN-α (177) and IL-12/23 (178). Deucravacitinib is FDA approved and currently under consideration by the European Medicines Agency for the treatment of moderate to severe psoriasis (179). To date, no preclinical or translational studies investigating the mechanism of action of deucravacitinib in AA have been published. Nevertheless, knowledge from psoriasis may substantiate potential efficacy in AA, specifically inhibition of IL-12 and IL-23 signaling through TYK2/JAK2. Both cytokines can be produced by myeloid dendritic cells upon activation by IFN-α released by pDCs, and both IL-12 and IL-23 play a key role in Th1 and Th17 differentiation, respectively (180). Each of these immune cell subsets has been described in AA, although the extent of the contribution of IL-12 and IL-23 needs to be investigated further (76, 98, 181, 182). A double-blind, randomized, placebo-controlled, phase 2 trial evaluating the safety and efficacy of 24 weeks of deucravacitinib treatment in adults with AA is currently recruiting (NCT05556265). Approximately 90 patients with AA and ≥50% scalp hair loss at baseline are expected to be enrolled. The primary endpoint will be change from baseline SALT score at week 24.

### 4.2 Dual-target JAK inhibitor: targeting JAK3 and TEC

As the underlying T-cell–mediated pathophysiology of AA may be multifactorial, rationale exists for targeting multiple signaling pathways involved (183). Recently approved by the FDA and EMA for treatment of severe AA in patients ≥12 years old, ritlecitinib is a selective dual inhibitor of the JAK3/TEC family of kinases—including TEC, Bruton tyrosine kinase (BTK), ITK, BMX, and TXK—binding irreversibly to JAK3 with >10,000-fold greater potency against JAK3 than JAK1, JAK2, and TYK2 (184, 185). JAK3 plays a key role in upregulation of the immunostimulatory cytokine IL-2 (119, 186), which regulates the opposing functions of effector cell response stimulation and maintenance of beneficial Tregs (187). Additionally, JAK3 does not inhibit IL-10, which potentially serves as an HF immune privilege guardian (184).

Through inhibition of the TEC family of kinases and JAK3, ritlecitinib may modulate several signaling pathways involved in the pathogenesis of AA, both in terms of cytokine and TCR signaling as reviewed earlier. Via inhibition and downregulation of JAK3, ritlecitinib has been shown in cellular assays and preclinical models to inhibit signaling of cytokines that drive effector cell activation and proliferation in AA, including IL-15 and the related positive feedback loop with IFN-γ (184, 185, 188, 189). Ritlecitinib also inhibits TEC kinases such as ITK, resulting in inhibition of cytolytic functions of NKG2D^+^CD8^+^ T cells (185, 188). Although ritlecitinib does not directly inhibit IFN-γ signaling involving JAK1/JAK2-pSTAT1 *in vitro* (184), it has been shown to reduce production of IFN-γ, possibly related to inhibition of TEC kinases and JAK3 (185, 188, 189).

Translational evidence from a phase 2a, randomized, double-blind, placebo-controlled trial of ritlecitinib in adults with AA (NCT04517864) was concordant with the reported mechanism of action (189, 190). In this study, blood concentrations of the chemokine IFN-γ–induced protein 10 (IP10; C-X-C motif chemokine ligand 10 [CXCL10]), which is induced by IFN-γ, were decreased at week 24 in patients receiving ritlecitinib; additionally, infiltration of inflammatory CD8^+^ and NKG2D^+^ T cells was reduced at the level of individual HFs (190). Treatment with ritlecitinib also resulted in broad transcriptomic changes extending beyond the Th1 axis, including changes to NK and T-cell activation, IL-12/23 levels, Th2, hair keratins, and hair keratin–associated proteins (189).

The ritlecitinib ALLEGRO phase 2b/3 trial (NCT03732807) examined efficacy and safety in patients aged ≥12 years with AA (26). Patients received once-daily ritlecitinib ± an initial 4-week loading dose: 200/50 mg, 200/30 mg, 50 mg, 30 mg, 10 mg (dose ranging only; not tested vs placebo), or placebo for 24 weeks. At 24 weeks, the primary endpoint was met by all active treatment groups; SALT score ≤20 response rates were 31%, 22%, 23%, 14%, and 2% in the 200/50-mg, 200/30-mg, 50-mg, 30-mg (*P*<0.001 for all), and placebo groups, respectively, and 2% in the 10-mg group (assessed for dose ranging only). These improvements were sustained or increased through week 48, reaching 43% and 40% in patients receiving ritlecitinib 50 mg 200/50 mg, respectively. The most frequent AEs reported among patients receiving ritlecitinib included nasopharyngitis, upper respiratory tract infection, headache, and acne. Serious infections, herpes zoster infections, MACE, and malignancies were infrequent.

### 4.3 Cytokine inhibitor

EQ101 (formerly BNZ-1) is a selective inhibitor of IL-2, IL-9, and IL-15 that targets the common γ-chain signaling receptor subunit shared by these cytokines, elevations of which have been reported in the serum of patients with AA (98, 191). Although IL-15 and IL-2 have been investigated in AA and are detailed in this review, no studies have specifically investigated the role of IL-9 in the pathophysiology of AA. IL-9 is known to take contrasting roles as a proinflammatory or immunosuppressive cytokine and has been associated with allergic diseases commonly co-occurring with AA (192). No preclinical or translational studies of EQ101 relevant to AA have been published to date, although EQ101 is currently under investigation in a phase 2 study for the treatment of AA with ≥50% scalp hair loss (NCT03532958). Pharmacodynamic results from a phase 1 trial of 18 healthy adults suggest that intravenously administered EQ101 doses of 0.5 to 1.6 mg/kg could result in prolonged reductions in the levels of CD8^+^ central memory T cells; total CD4^+^ and CD8^+^ T cells, B cells, and monocyte levels were not changed as a result of EQ 101 exposure (191). No dose-limiting toxicities, infusion reactions, or serious AEs were observed among patients receiving EQ101 in this phase 1 study of 18 healthy adults (191). A phase 2 open-label study evaluating the safety, efficacy, pharmacokinetics, and pharmacodynamics of 24 weeks of treatment with EQ101 in 30 adult patients with AA and >35% scalp hair loss is currently planned, with a primary endpoint of incidence of treatment-emergent AEs through 28 weeks (NCT05589610).

### 4.4 Inhibition of T-cell migration

S1P modulation has been validated in autoimmune diseases (193), and S1P agonists are approved in multiple regions, including the US and Europe, to treat multiple sclerosis (194). Etrasimod is an S1P receptor modulator currently under investigation for the treatment of T-cell–mediated inflammatory conditions, including AA. S1P modulation with etrasimod is hypothesized to lead to lymphocyte sequestration in skin-draining lymph nodes, preventing lymphocyte migration to the HF, similar to the lymphocyte egress observed with drugs targeting S1P in other inflammatory diseases (195, 196). A phase 2 study of the safety and efficacy of etrasimod in patients with AA and ≥25% to <95% scalp hair loss is currently ongoing and scheduled for completion in October 2023 (NCT04556734). The primary endpoint will be percent change from baseline in SALT score at week 24.

### 4.5 Inhibition of ILT7

Leukocyte immunoglobulin-like receptor subfamily A member 4 (ILT7) is an Ig-like cell surface receptor highly expressed in pDCs. ILT7 signaling in pDCs has implications for TLR response and downregulation of downstream IFN-α production (63), which is hypothesized to contribute to the early phase of AA through priming and differentiation of T cells and stimulation of IFN-γ release. Daxdilimab is an antibody targeting ILT7 and is currently under development for several inflammatory conditions, including AA. Binding of ILT7 with daxdilimab may result in the depletion of pDCs through antibody-dependent cellular cytotoxicity and/or inhibition of the production of IFN-α through agonistic effect, although no data regarding the mechanism of action have been published to date. A phase 2 trial assessing the safety, efficacy, tolerability, pharmacokinetics, and pharmacodynamics of daxdilimab in adults with AA and 50% to 95% scalp hair loss is currently recruiting (NCT05368103). The primary endpoint is percent change from baseline in SALT score at week 24.

## 5 Perspectives and future directions

As the importance of T cells in the autoimmune pathogenesis of AA is increasingly understood, several therapeutic strategies aimed at reducing T-cell signaling, migration, proliferation, and activity have emerged or are under development. Given the multifactorial nature of the signaling pathways that underlie the immune-mediated attack of the HF in AA, there is a rationale to develop therapies that target multiple distinct signaling pathways beyond the JAK/STAT pathway first considered for therapeutic development. Precedent for a dual-targeted therapeutic approach exists for the treatment of inflammatory bowel disease, for which several targeted therapies are available (197). Dual-targeted therapies using either a combination of drugs or a single drug with a dual mechanism of action hold promise to overcome the ceiling effect of current therapies, drug resistance, or immune escape (197).

In the future, other T-cell–related pathways beyond those discussed may serve as targets for therapeutic development in AA. Three approaches have been explored in recent years: targeting disease-promoting mediators, promoting immune tolerance to reestablish immune homeostasis, and targeting the microbiome.

The most conventional of these approaches is the direct targeting of disease-promoting mediators. Although NKG2D is a well-known activator of the immune system in response to “induced self” ligands and is implicated in the autoimmune pathogenesis of AA, no current therapeutics targeting NKG2D are currently under investigation in AA (198). One trial of the safety and efficacy of the anti-NKG2D antibody tesnatilimab (formerly known as JNJ-64304500) in patients with AA (NCT04740970) has been withdrawn per sponsor decision. However, therapies targeting NKG2D are under investigation in other autoimmune diseases such as rheumatoid arthritis and Crohn disease (198), and NKG2D^+^CD8^+^ T cells have been implicated in vitiligo (199).

CXCR3 is another receptor expressed by activated CD8^+^ T cells (200). In a preclinical study using a murine graft model of AA, inhibition of CXCR3 with antibodies was shown to prevent the development of AA by inhibiting the accumulation of NKG2D^+^CD8^+^ T cells in the cutaneous lymph nodes and skin (53). These preliminary results suggest that interference with the cytotoxic T cell 1 response by inhibiting CXCR3 could represent a viable therapeutic strategy for prevention or treatment of AA (53).

PD-1 is an immunoreceptor of the CD28/CTLA-4 family that regulates antigen receptor signaling and protects against autoimmunity by promoting apoptosis of antigen-specific T cells while reducing apoptosis in Tregs (201–203). Cell-based studies suggest that PD-1 modulates IFN-γ secretion (136); further, JAK1/3 inhibition has been shown to increase PD-1 expression (166). As PD-1 is thought do downregulate T-cell–mediated immune responses, PD-1 agonism is hypothesized to broadly suppress inflammatory diseases including AA (201). Rosnilimab (formerly ANB303) is a humanized IgG1 PD-1 agonistic antibody examined recently in a phase 2 study of patients with AA and ≥50% scalp hair loss (NCT05205070). The investigating company announced in early 2003 that further development of rosnilimab in AA will not be pursued due to lack of efficacy on hair regrowth as evaluated by SALT score in this trial.

Targeting immune tolerance to attenuate inflammatory processes is an emerging but conceptual approach for the possible treatment of AA. Self-antigens located in the healthy lower anagen HF, the target site in AA, are likely to interact with T cells in an unusual way, including inducing a form of tolerance to normally rejected stimuli (204). With additional research, strategies that upregulate TGF-β signaling, modulate T-cell function through TLRs, reduce autoantigen-specific T-cell counts, or improve beneficial Treg activity could represent viable therapeutic approaches in the future (205). Indeed, a related approach aimed at inducing beneficial Tregs is under consideration for the treatment of multiple sclerosis (206).

With the advent of CD19-reactive chimeric antigen receptor (CAR) T-cell therapy for treatment of B-cell lymphoma (207), there is growing interest in the potential use of engineered antigen-specific Tregs in conjunction with IL-2 for the treatment of autoimmune skin conditions, including AA (208). Similarly, the potential to tolerize/immunize with HF autoantigens in AA has been previously suggested both conceptually (209) and from experimental data (210) showing that vaccination with soluble hair-specific keratin peptides can significantly delay AA induction and prevent progression. Although reduced Treg activity could factor into immune privilege collapse at the HF, it is unclear whether Tregs play a primary or secondary role in AA. For example, attempts to reverse AA in a mouse model and in humans by inducing Tregs with IL-2 have been unsuccessful to date (154, 155). An *in vitro* study of IL-2 mutant proteins engineered to preferentially stimulate Tregs over CD4^+^ or CD8^+^ T cells is planned (NCT05544448). The effectiveness of these approaches alone, or in combination or following treatment targeting T-cell signaling, also remains to be evaluated. Overall, targeting innate immunity and self-tolerance remains a relatively nascent and underexplored avenue of therapeutic development.

Finally, as relationships between the microbiome and autoimmunity are increasingly studied (211), modulation of the microbiome may represent a future therapeutic strategy for AA. Dysbiosis has been identified both on the scalp (212) and in the gut (213) of patients with AA, suggesting a possible relationship between AA and the microbiome. While evidence of a direct connection between the microbiome and AA remains uncertain, a case report of 2 patients with alopecia totalis reported hair regrowth after a fecal microbiota transplant for treatment of *C. difficile* infections (214).

## 6 Conclusions

Therapies focused on targeting T-cell signaling activity are a promising strategy for the management of AA. Although outcomes achieved in clinical trials evaluating JAK inhibitors for AA have been positive, there have always been nonresponders, implying the complexity of AA immunology and reinforcing the need for multiple therapeutic remedies targeting a variety of pathways involved in the pathophysiology of this disease. AA by nature is often a chronic and relapsing disease (3, 17, 78, 215), and a shifting repertoire of T-cell specificities to an ever-expanding repertoire of follicular autoantigens that emerge from repeated cycles of HF tissue disruption will further dictate the development of therapeutics targeting pathways beyond JAK/STAT signaling. This is especially true for patients with severe AA who may require multiple therapeutics with distinct mechanisms of action to achieve disease control over time with good long-term tolerance.

As the pathogenesis of AA is better understood, molecular profiling of disease markers before and after therapy could lead to better control of disease in the future. Molecular and cellular characterization of treatment responders and nonresponders may allow for a clearer definition of phenotypes or endotypes of AA, may better explain differential response to therapy across different body areas within the same patient, and may assist in the development and application of immune-based therapies targeted to the specific signaling pathways involved in AA. The near future will illuminate new strategies for targeting and modulating specific aspects of T-cell signaling to improve efficacy, safety, and tolerability of AA treatments.

## Author contributions

All authors listed have made a substantial, direct, and intellectual contribution to the work and approved it for publication.
